# Supplementary material for: ACBD3 Is an Essential Pan-enterovirus Host Factor That Mediates the Interaction between Viral 3A Protein and Cellular Protein PI4KB
Source: mBio. 2019 Feb 12;10(1):e02742-18. doi: 10.1128/mBio.02742-18 (PMC6372799; doi:10.1128/mBio.02742-18)
Supplement: FIG S1 [file mBio.02742-18-sf001.pdf]

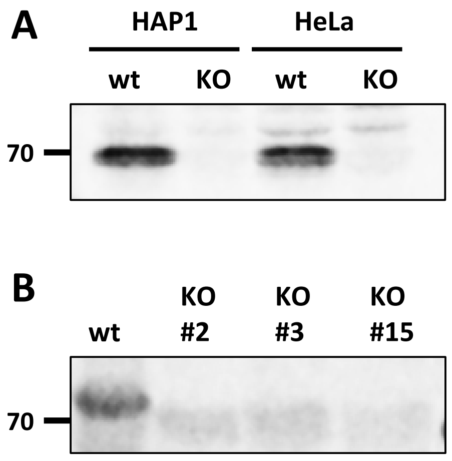

Figure S1. Generation of knockout cells using the CRISPR-Cas9 system.
The knockout of ACBD3 in HAP1 and HeLa cells (A) and PI4KB in HeLa cells (three individual clones) (B) was confirmed by Western blot analysis of cell lysates.
