# Supplementary material for: ACBD3 Is an Essential Pan-enterovirus Host Factor That Mediates the Interaction between Viral 3A Protein and Cellular Protein PI4KB
Source: mBio. 2019 Feb 12;10(1):e02742-18. doi: 10.1128/mBio.02742-18 (PMC6372799; doi:10.1128/mBio.02742-18)
Supplement: FIG S2 [file mBio.02742-18-sf002.pdf]

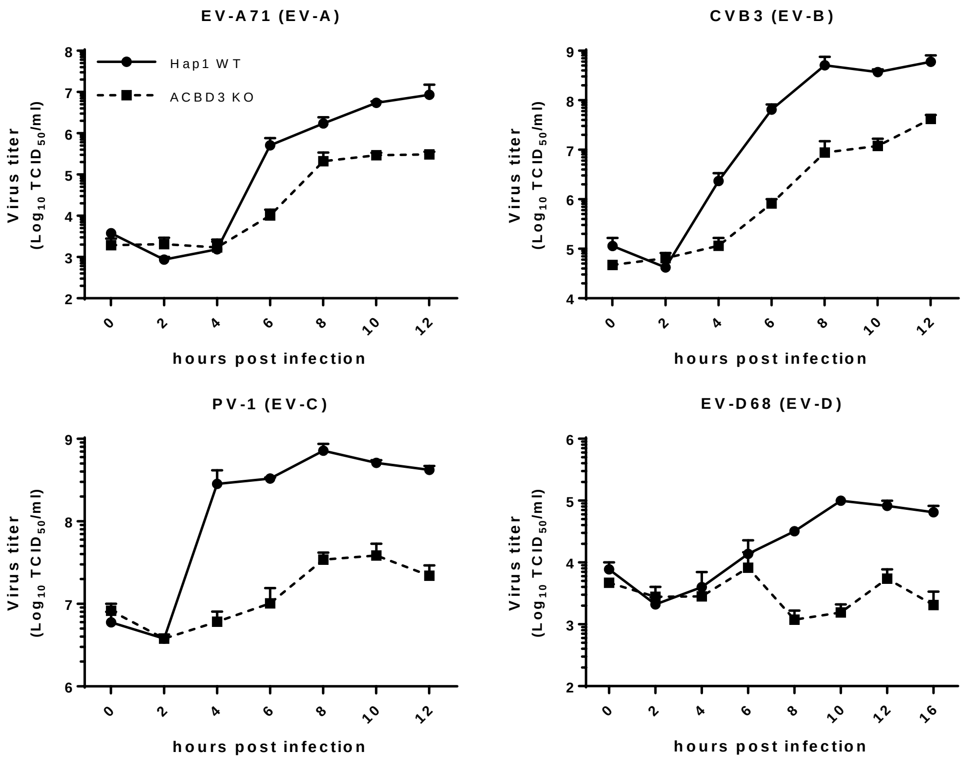

Figure S2. Enterovirus replication is inhibited in HAP1 ACBD3^{KO} cells.

Growth curves of enteroviruses in HAP1^{wt} and ACBD3^{KO} cells. After infection at an MOI 3-5 for 30 min, the inoculum was removed and fresh medium was added to the cells. At the indicated time points, cells were freeze-thawed to determine the total virus titers by endpoint dilution.
