# Supplementary material for: ACBD3 Is an Essential Pan-enterovirus Host Factor That Mediates the Interaction between Viral 3A Protein and Cellular Protein PI4KB
Source: mBio. 2019 Feb 12;10(1):e02742-18. doi: 10.1128/mBio.02742-18 (PMC6372799; doi:10.1128/mBio.02742-18)
Supplement: FIG S3 [file mBio.02742-18-sf003.pdf]

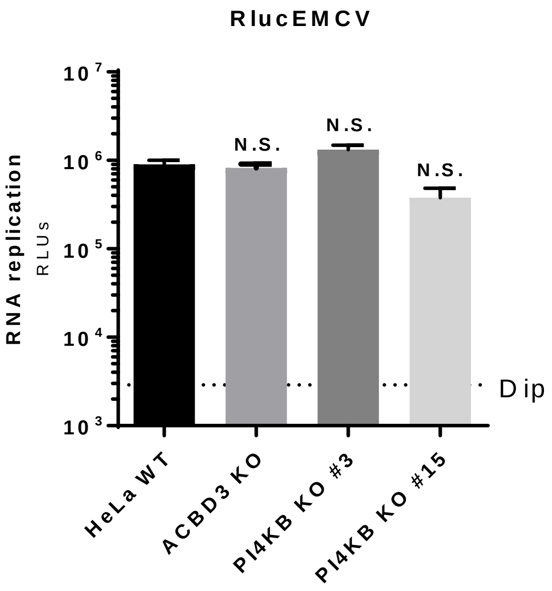

Figure S3. EMCV replication is not sensitive to ACBD3 or PI4KB depletion.

HeLa^{wt}, ACBD3^{KO}, and PI4KB^{KO} cells were infected with wt EMCV reporter virus carrying a Renilla luciferase (RlucEMCV) at an MOI 0.1. After 8 h, cells were lysed to determine luciferase activity. Bars represent the mean of triplicate values ± SEM. Dip: Dipyridamole, an inhibitor of EMCV replication. Values were statistically evaluated compared to the values of HeLa^{wt} cells using a one-way ANOVA. N.S., not significant.
