# Supplementary material for: ACBD3 Is an Essential Pan-enterovirus Host Factor That Mediates the Interaction between Viral 3A Protein and Cellular Protein PI4KB
Source: mBio. 2019 Feb 12;10(1):e02742-18. doi: 10.1128/mBio.02742-18 (PMC6372799; doi:10.1128/mBio.02742-18)
Supplement: FIG S4 [file mBio.02742-18-sf004.pdf]

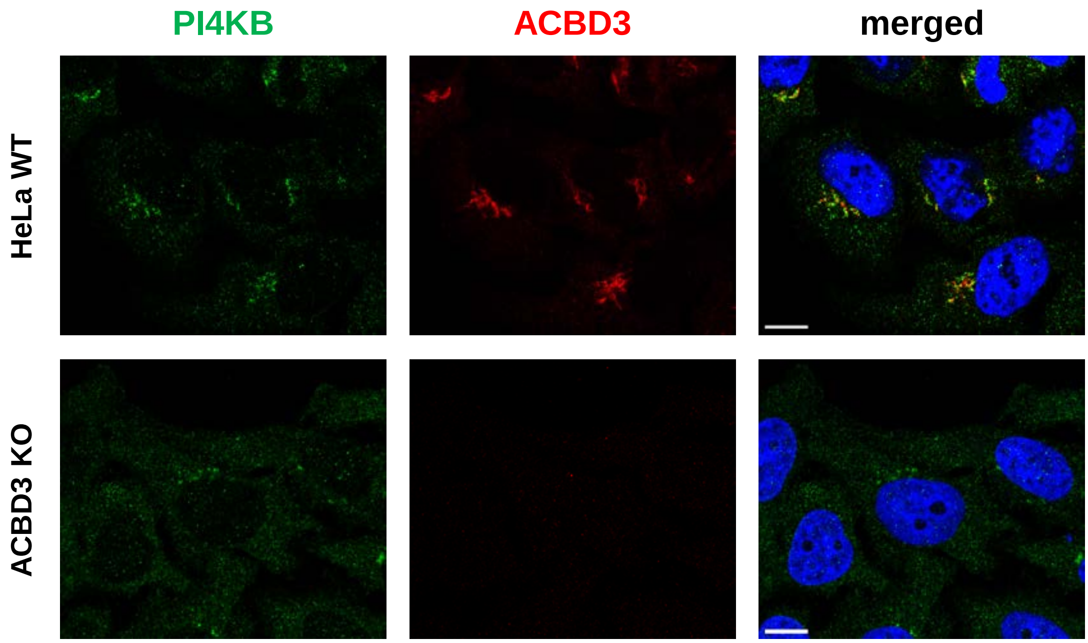

Figure S4. Localization of PI4KB in HeLa^{wt} and ACBD3^{KO} cells.

HeLa^{wt} and ACBD3^{KO} cells were fixed and stained with antibodies against PI4KB (green) and ACBD3 (red). Nuclei were stained with DAPI (blue). Scale bars represent 10 μm.
