# Supplementary material for: ACBD3 Is an Essential Pan-enterovirus Host Factor That Mediates the Interaction between Viral 3A Protein and Cellular Protein PI4KB
Source: mBio. 2019 Feb 12;10(1):e02742-18. doi: 10.1128/mBio.02742-18 (PMC6372799; doi:10.1128/mBio.02742-18)
Supplement: FIG S5 [file mBio.02742-18-sf005.pdf]

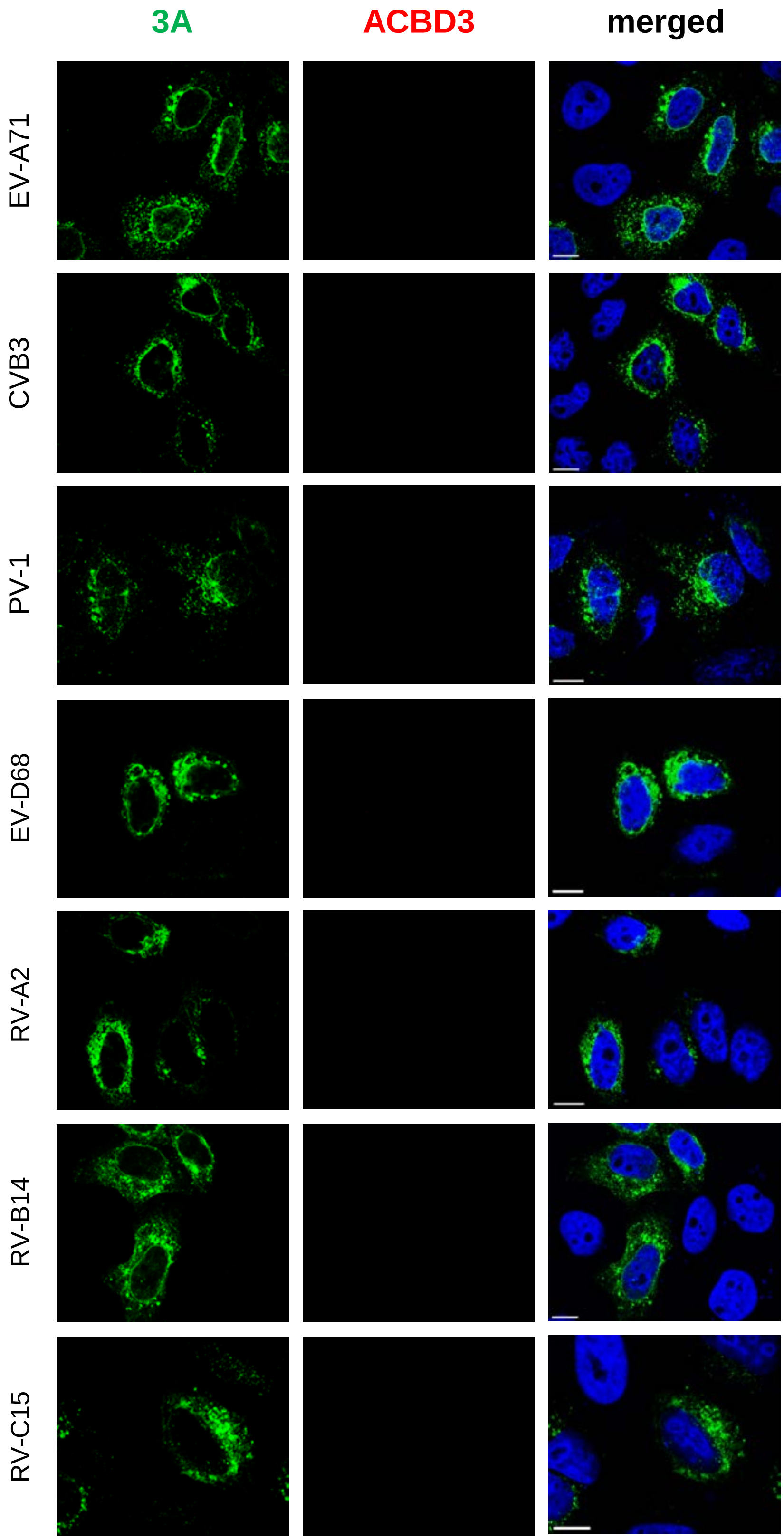

Figure S5. Localization of enterovirus 3A proteins in ACBD3^{KO} cells.

HeLa ACBD3^{KO} cells were transfected with plasmids encoding myc-tagged EV-A71 3A, CVB3 3A, PV-1 3A, or EGFP-tagged EV-D68 3A, RV-2 3A, RV-14 3A. The next day, cells were fixed and stained with antibodies against the myc tag to detect 3A (green) and ACBD3 (red). Nuclei were stained with DAPI (blue). Asterisks indicate 3A expressing cells. Scale bars represent 10 µm.
