# Supplementary material for: ACBD3 Is an Essential Pan-enterovirus Host Factor That Mediates the Interaction between Viral 3A Protein and Cellular Protein PI4KB
Source: mBio. 2019 Feb 12;10(1):e02742-18. doi: 10.1128/mBio.02742-18 (PMC6372799; doi:10.1128/mBio.02742-18)
Supplement: FIG S6 [file mBio.02742-18-sf006.pdf]

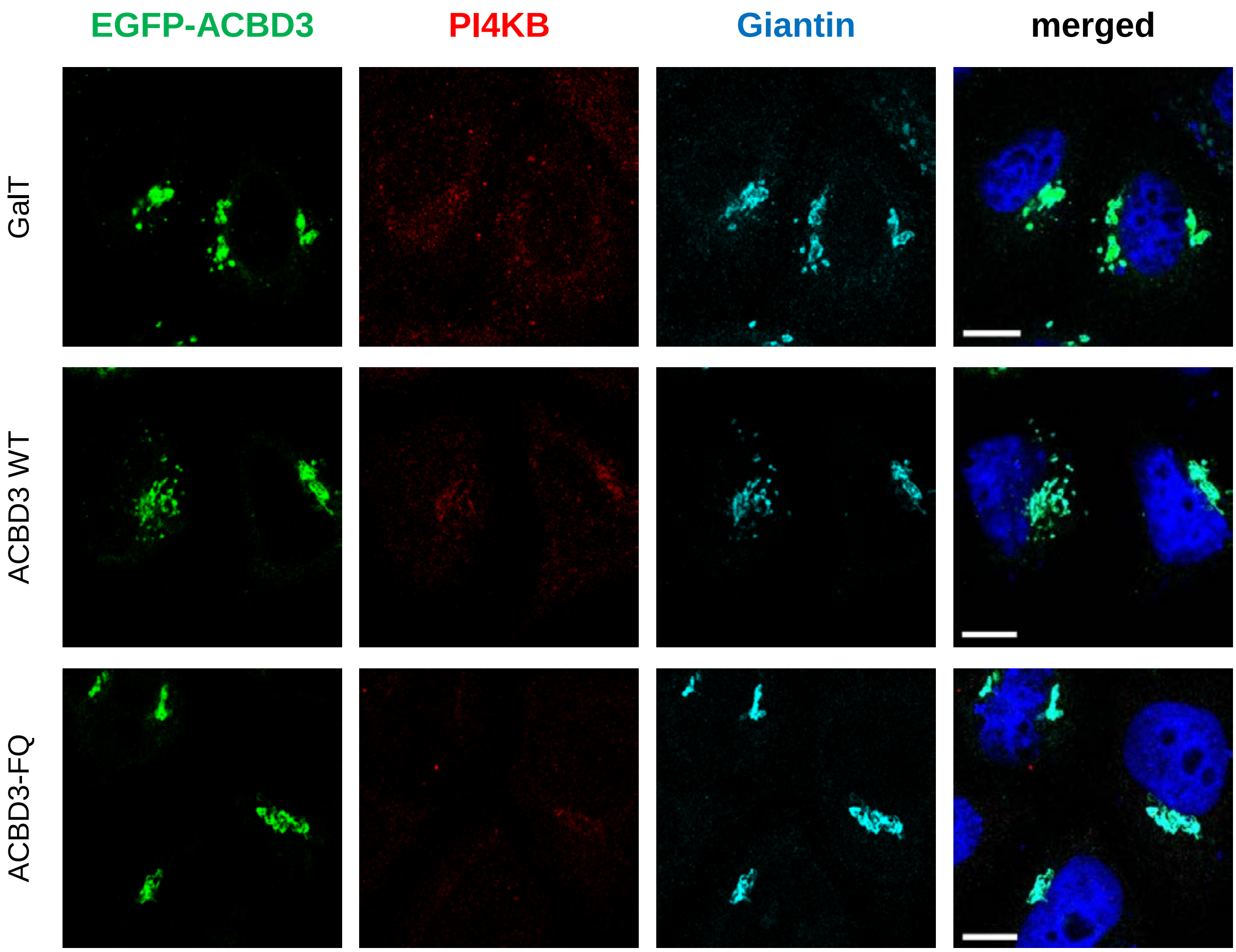


**Figure S6. Effects of ACBD3 reconstitution on PI4KB localization in ACBD3$^{KO}$ cells.**
HeLa ACBD3$^{KO}$ cells were transfected with plasmids encoding EGFP-tagged GalT, ACBD3 wt, or ACBD3-FQ mutant. The next day, cells were fixed and stained with the antibodies against PI4KB (red) and Giantin (light blue). Nuclei were stained with DAPI (blue). Scale bars represent 10 μm.
