# Supplementary material for: ACBD3 Is an Essential Pan-enterovirus Host Factor That Mediates the Interaction between Viral 3A Protein and Cellular Protein PI4KB
Source: mBio. 2019 Feb 12;10(1):e02742-18. doi: 10.1128/mBio.02742-18 (PMC6372799; doi:10.1128/mBio.02742-18)
Supplement: FIG S7 [file mBio.02742-18-sf007.pdf]

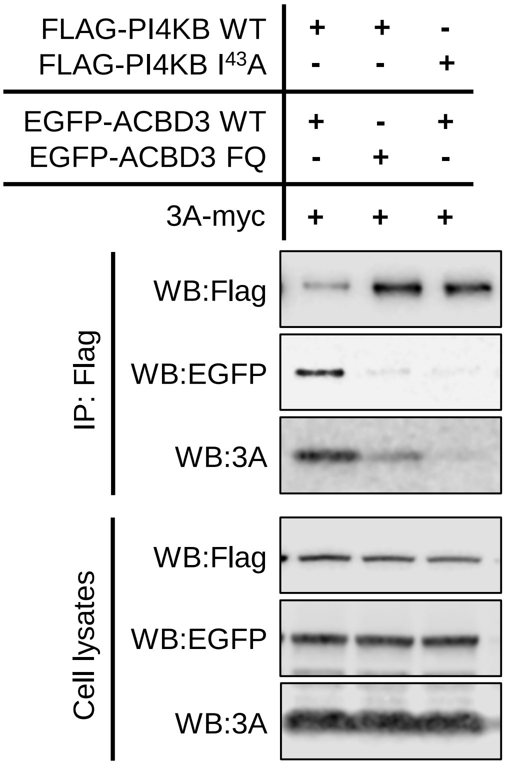

Figure S7. Co-immunoprecipitation of PI4KB with ACBD3 and enterovirus 3A protein.
Enterovirus 3A has been reported to enhance the interaction between ACBD3 and PI4KB (Xiao *et al.*, 2017). We therefore evaluated the ACBD3-PI4KB interaction by co-immunoprecipitation in the presence of CVB3 3A. HEK293T cells were co-transfected with plasmids encoding FLAG-tagged PI4KB wt or I43A mutant, EGFP-tagged ACBD3 wt or FQ mutant, and myc-tagged CVB3 3A. Immunocomplexes were captured by anti-FLAG beads and subjected to Western blot analysis. A PI4KB mutant (I43A) and an ACBD3 mutant (FQ), which have reduced interaction between PI4KB-ACBD3 in vitro (Klima *et al.*, 2016; McPhail *et al.*, 2017), clearly disrupted this interaction in cells as well. As CVB3 3A was co-immunoprecipitated with wt PI4KB and ACBD3 but not with the mutants, we confirmed the formation of an enteroviral 3A-ACBD3-PI4KB ternary complex in which ACBD3 links 3A and PI4KB, which is in agreement with previous findings for EV-A71 (Xiao *et al.*, 2017). Of note, we repeatedly observed less immunoprecipitation of wt PI4KB when the 3A-ACBD3-PI4KB ternary complex was formed (first lane), which suggests that the binding of 3A-ACBD3 to the N-terminal part of PI4KB interferes with binding of the complex to the anti-FLAG beads.
