# Supplementary material for: ACBD3 Is an Essential Pan-enterovirus Host Factor That Mediates the Interaction between Viral 3A Protein and Cellular Protein PI4KB
Source: mBio. 2019 Feb 12;10(1):e02742-18. doi: 10.1128/mBio.02742-18 (PMC6372799; doi:10.1128/mBio.02742-18)
Supplement: TEXT S1 [file mBio.02742-18-s0001.pdf]

Supplemental Material:

Immunoprecipitation

HEK293T cells were grown on 150 mm dishes. Subconfluent cells were transfected with in total 15 ug of plasmids using Lipofectamine 2000 (Thermo Fisher ScientificThermo) according to the manufacturer's protocol. At 24 h post transfection, cells were harvested and lysed by using lysis buffer (50 mM TrisHCl pH 7.4, 150 mM NaCl, 1 mM EDTA, 1 mM DTT, 10% Glycerol, 1% Triton X-100). After 30 min incubation on ice, lysates were centrifuged for 20 min at 10,000 xg. Supernatants were incubated with anti-FLAG M2 magnetic beads (Sigma) at 4°C for 2 h. Beads were washed 3 times with the lysis buffer and then incubated with Laemmli sample buffer for 5 min at 95°C. Eluted proteins were subjected to Western blot analysis.
