# Supplementary material for: ACBD3 Is an Essential Pan-enterovirus Host Factor That Mediates the Interaction between Viral 3A Protein and Cellular Protein PI4KB
Source: mBio. 2019 Feb 12;10(1):e02742-18. doi: 10.1128/mBio.02742-18 (PMC6372799; doi:10.1128/mBio.02742-18)
Supplement: TEXT S2 [file mBio.02742-18-s0002.pdf]

Supplemental references:

Xiao X, Lei X, Zhang Z, Ma Y, Qi J, Wu C, Xiao Y, Li L, He B, Wang J. 2017. Enterovirus 3A facilitates viral replication by promoting PI4KB-ACBD3 interaction. J Virol. 91:e00791-17

Klima M, Toth DJ, Hexnerova R, Baumlova A, Chalupska D, Tykvart J, Rezabkova L, Sengupta N, Man P, Dubankova A, Humpolickova J, Nencka R, Veverka V, Balla T, Boura E. 2016. Structural insights and in vitro reconstitution of membrane targeting and activation of human PI4KB by the ACBD3 protein. Sci Rep 6:23641.

McPhail JA, Ottosen EH, Jenkins ML, Burke JE. 2017. The Molecular Basis of Aichi Virus 3A Protein Activation of Phosphatidylinositol 4 Kinase IIIbeta, PI4KB, through ACBD3. Structure 25:121-131.
